# A Systematic Review and Meta-Analysis of Phytoestrogen Protects Against Myocardial Ischemia/Reperfusion Injury: Pre-Clinical Evidence From Small Animal Studies

**DOI:** 10.3389/fphar.2022.847748

**Published:** 2022-05-20

**Authors:** Yumeng Wang, Xintian Shou, Zongjing Fan, Jie Cui, Donghua Xue, Yang Wu

**Affiliations:** ^1^ Graduate School, Beijing University of Chinese Medicine, Beijing, China; ^2^ Department of Cardiovascular, Dongfang Hospital, Beijing University of Chinese Medicine, Beijing, China; ^3^ Department of Cardiovascular, Guang’anmen Hospital, China Academy of Chinese Medical Sciences, Beijing, China

**Keywords:** phytoestrogen, isoflavones, myocardial ischemia reperfusion injury, molecular mechanisms, preclinical, meta-analysis, systematic review

## Abstract

**Background:** Phytoestrogens are a class of natural compounds that have structural similarities to estrogens. They have been identified to confer potent cardioprotective effects in experimental myocardial ischemia-reperfusion injury (MIRI) animal models. We aimed to investigate the effect of PE on MIRI and its intrinsic mechanisms.

**Methods:** A systematic search was conducted to identify PEs that have been validated in animal studies or clinical studies as effective against MIRI. Then, we collected studies that met inclusion and exclusion criteria from January 2016 to September 2021. The SYRCLE’s RoB tool was used to evaluate the quality. Data were analyzed by STATA 16.0 software.

**Results:** The search yielded 18 phytoestrogens effective against heart disease. They are genistein, quercetin, biochanin A, formononetin, daidzein, kaempferol, icariin, puerarin, rutin, notoginsenoside R1, tanshinone IIA, ginsenoside Rb1, ginsenoside Rb3, ginsenoside Rg1, ginsenoside Re, resveratrol, polydatin, and bakuchiol. Then, a total of 20 studies from 17 articles with a total of 355 animals were included in this meta-analysis. The results show that PE significantly reduced the myocardial infarct size in MIRI animals compared with the control group (*p* < 0.001). PE treatment significantly reduced the creatine kinase level (*p* < 0.001) and cTnI level (*p* < 0.001), increased left ventricular ejection fraction (*p* < 0.001) and left ventricular fractional shortening (*p* < 0.001) in MIRI animals. In addition, PE also exerts a significant heart rate lowering effect (*p* < 0.001).

**Conclusion:** Preclinical evidence suggests that PE can be multi-targeted for cardioprotective effects in MIRI. More large animal studies and clinical research are still needed in the future to further confirm its role in MIRI.

## 1 Introduction

Ischemic heart diseases, such as coronary artery disease (CHD), represent the most the leading cause of death worldwide (Ibáñez et al., 2015; Hausenloy et al., 2017), which has crucial socio-economical implications. Percutaneous coronary intervention (PCI) is the primary means of revascularization in patients with coronary artery disease (O'Gara et al., 20132013). Timely and effective PCI treatment can recanalize the occluded coronary artery, reestablish blood perfusion in the infarcted area, and salvage ischemic myocardial tissue, which improves the survival rate of patients with coronary heart disease (Hausenloy and Yellon, 2013). However, it can also paradoxically cause further myocardial ischemia-reperfusion injury (MIRI), which can manifest clinically as an increase in infarct size, cardiac insufficiency, myocardial stunning, arrhythmias, and even sudden death (Piper et al., 1998).

Although, several therapies have been approved to give cardioprotection in experimental models of MIRI (Mokhtari-Zaer et al., 2018; Sawashita et al., 2020; Sun et al., 2021; Wu et al., 2021). MIRI is currently not treated in a clinically effective manner. Estrogen is known to perform well in cardioprotection (Knowlton and Lee, 2012; Crescioli, 2021). But this cardiovascular protection declines after menopause, with myocardial infarction being the primary cause of mortality in older women (Wenger, 2016). Estrogen deficiency has been shown to play an important role in the development of cardiovascular diseases such as MIRI (Deschamps et al., 2010; Sivasinprasasn et al., 2016), atherosclerosis (Kassi et al., 2015; Hajializadeh and Khaksari, 2021), heart failure ((Hajializadeh and Khaksari, 2021), (Adekunle et al., 2021)), atrial fibrillation (Bretler et al., 2012; Odening et al., 2019), hypertension (Colafella and Denton, 2018; Srivaratharajah and Abramson, 2019), myocardial fibrosis (Medzikovic et al., 2019), cardiac hypertrophy (Hajializadeh and Khaksari, 2021), and Takotsubo syndrome (Pelliccia et al., 2017). However, the use of estrogen or estrogen replacement therapy for an extended period can raise the risk of gynecological cancers (Stevenson et al., 2009; Narod, 2011).

Phytoestrogens (PEs) are a class of natural non-steroidal compounds widely found in many plants and herbs (Sirotkin and Harrath, 2014; Rietjens et al., 2017). It can bind to estrogen receptors (ER) to exert estrogen-like effects with few side effects (Kurzer and Xu, 1997; Sirotkin and Harrath, 2014; Li et al., 2016a; Basu and Maier, 2018). PE and PE-containing drugs have been shown to help prevent and treat menopausal symptoms (Franco et al., 2016), osteoporosis (Thangavel et al., 2019), metabolic diseases (Mukund et al., 2017), and especially cardiovascular diseases (Frankenfeld, 2017; Kirichenko et al., 2017; Sathyapalan et al., 2018). Notably, PEs have been identified that confer robust cardioprotection in experimental MIRI animal and cellular models through multiple molecular pathway modalities, such as inflammatory pathways, mitochondrial energy metabolic pathways, oxidative stress pathways, autophagic pathways, etc (Ji et al., 2004; Guo et al., 2018; Huang et al., 2018; Bai et al., 2019; Colareda et al., 2020).

PEs have become a promising cardioprotective candidate for MIRI due to their advantages of multiple therapeutic targets, lower side effects, and higher safety compared to estrogen replacement therapy (Sirtori, 2001; Bloedon et al., 2002; Michael McClain et al., 2006; Douglas et al., 2013; Czuczwar et al., 2017; Bolton et al., 2019).

Systematic preclinical study review aids in the elucidation of pharmacological mechanisms, the integration of therapeutic evidence, the improvement of experimental methods, and, eventually, the translation and transformation from animal studies to clinical trials (Roberts et al., 2002; Sandercock and Roberts, 2002). This study aims to elucidate the treatment mechanism of PE on MIRI and provide credible preclinical evidence by conducting a systematic review.

## 2 Methods

### 2.1 Identification of PEs

A systematic search of four databases (Pubmed, Web of Science, Embase, and Cochrane Library) was conducted to identify PEs that have been validated in animal studies or clinical studies for interventions in MIRI.

### 2.2 Search Strategy

**FIGURE 1:**
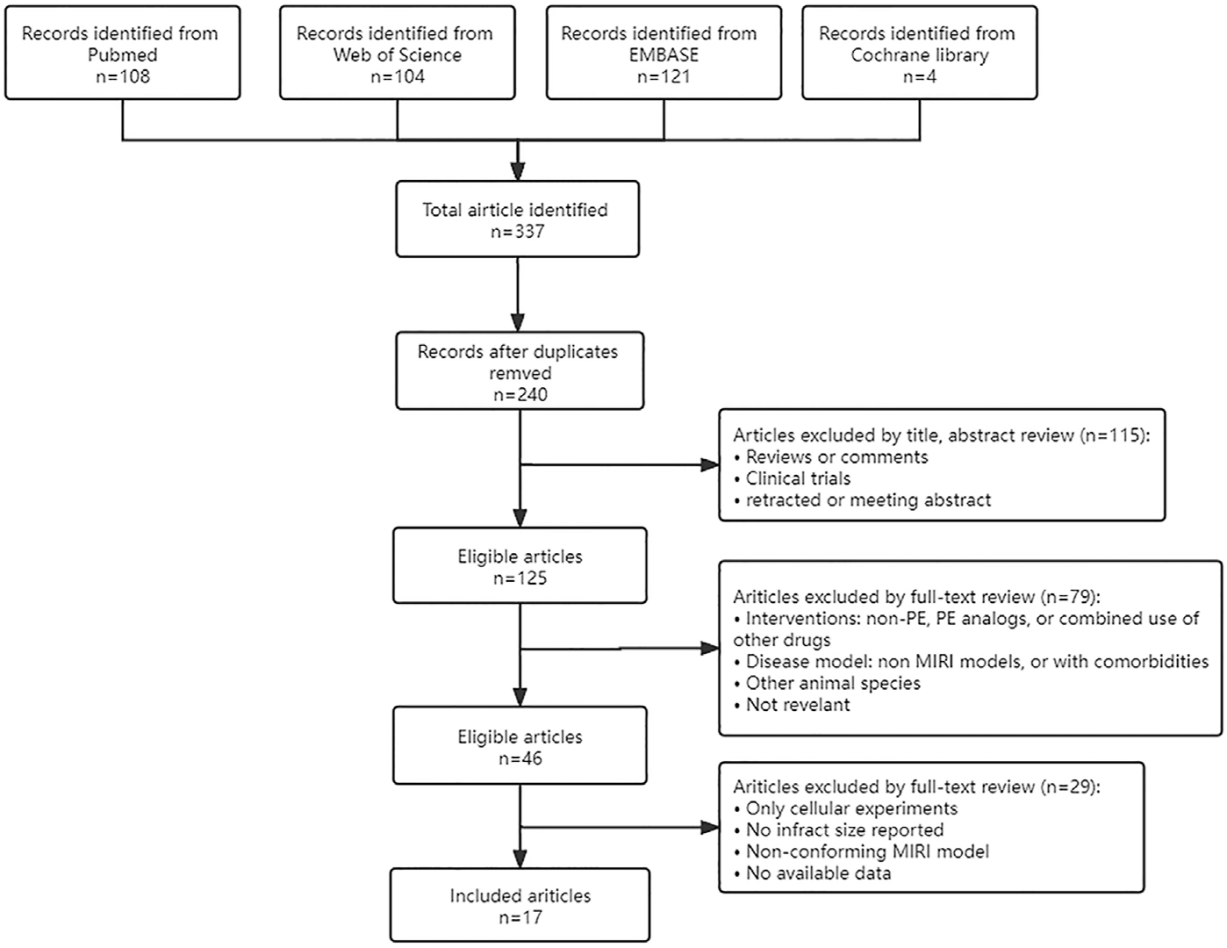
Flow chart of records retrieved, screened and included in this meta-analysis.

Four databases (Pubmed, Web of Science, Embase, and Cochrane Library) were searched using “myocardial ischemia-reperfusion injury”, “myocardial reperfusion injury”, and the PE retrieved in 2.1 as keywords. Then manually search and add any literature that may have been missed. Timespan: January 2016 - September 2021. Only published articles written in the English language were considered in the current meta-analysis. The specific search process was shown in Figure 1.

### 2.3 Inclusion and Exclusion Criteria

The following criteria were set in advance. Inclusion criteria: 1) Rats or mice were used as research subjects; 2) methods for establishing animal models of MIRI: ligation and loosening surgery of the left anterior descending branch of coronary artery (*in vivo*), or Langendroff perfusion (*ex vivo*); 3) the treatment group received any dose of PE, while the control group received vehicle or no treatment; 4) The myocardial infarction size was taken as the main outcome index, with or without other indexes such as myocardial enzymes, heart rate (HR), left ventricular ejection fraction (LVEF), and left ventricular fractional shortening (LVFS); 5) the size of the myocardial infarction is reported as a percentage (i.e. TTC staining or Evan’s blue/TTC staining). Exclusion criteria: 1) animals with other cardiovascular comorbidities (e.g. diabetes, hyperlipidemia); 2) animals are treated with PE analogs or given additional drugs. 3) Studies with incomplete and inaccessible data; 4) duplicate publications.

### 2.4 Study Characteristics Extraction

Two authors (YM W and XT S) extracted the study characteristics independently, and discrepancies were resolved by the corresponding author. Information extracted included: first author, year of publication, anesthetics, animal information (animal species, number, sex, and weight), I/R duration, interventions (PE type, dose, method of administration) for the treatment and control groups, outcome indicators, and staining method of infarct size. The data were pooled using the formula as follows when different doses of pharmacological interventions were employed in the treatment group (Zhang and Chen, 2016). When data are represented graphically, every effort will be made to contact the author for more information or to measure from the graph.

$$\sqrt{\frac{\sum_{i=1}^{m}\left(n_{i}-1\right)SD_{i}^{2}+\sum_{i=1}^{m}n_{i}{\left({\bar{x}}_{i}-{\bar{x}}_{T}\right)}^{2}}{\sum_{i=1}^{m}\left(n_{i}-1\right)}}$$

### 2.5 Quality Appraisal

The SYRCLE’s RoB tool (Hooijmans et al., 2014) was used to evaluate the quality of the studies by two authors independently. The SYRCLE’s RoB tool contains 10 entries: sequence generation, baseline characteristics, allocation concealment, random housing, blinding, random outcome assessment, blinding, incomplete outcome data, selective outcome reporting, and other sources of bias. A third person ruled in case of disagreement.

### 2.6 Outcome Measures and Statistical Analyses

Data were analyzed by STATA 16.0 software. Outcomes were expressed as standardized mean difference (SMD) with a 95% confidence interval (95%CI). In the forest plot, the dark squares represent the standardized mean difference (SMD) for each study, the diamonds represent the pooled SMD, and 95% of the CIs are indicated by lines. *p* values ≤0.05 were considered statistically significant. Statistics were analyzed using a fixed-effects model (I^2^ ≤ 50%, *p* ≥ 0.10) or a random-effects model (I^2^ > 50%, *p* < 0.10). Sensitivity analysis, stratification analysis, or univariable meta-regression should be performed to deal with high heterogeneity if necessary. We performed sensitivity analysis by removing each study in turn to assess the impact of this study. We conducted a stratified analysis and meta-regression of myocardial infarction size by study type, route of administration, staining method, PE type, animal species, and anesthetics type. The Egger’s test and Begg’s test for publication bias were performed using STATA 16.0.

## 3 Results

### 3.1 Identification of PEs

The following PEs were retrieved that have been experimentally proven to be effective against MIRI. They are divided into four main categories: 1) Isoflavones: genistein, quercetin, biochanin A, formononetin, daidzein, kaempferol, icariin, puerarin, rutin, et al.; 2) Terpenoids: notoginsenoside R1, tanshinone IIA, ginsenoside Rb1, ginsenoside Rb3, ginsenoside Rg1, ginsenoside Re, et al.; 3) stilbenoids: resveratrol, polydatin, et al.; 4) miscellaneous classes: bakuchiol. We collected the chemical structure of these PEs and the representative herbs. The specific information is shown in Table 1.

**TABLE 1:** Phytoestrogens that have been experimentally proven to be effective against MIRI.

Type	PE	CAS	SD Structure^1^	Representative herbs^2^
Isoflavones	genistein	446-72-0	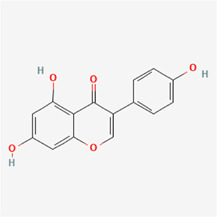	*Radix Puerariae, Spatholobus Suberectus Dunn*
	quercetin	117-39-5	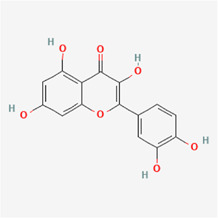	*Hedysarum Multijugum Maxim., Carthami Flos, Panax Notoginseng (Burk.) F. H. Chen Ex C. Chow*
	biochanin A	491-80-5	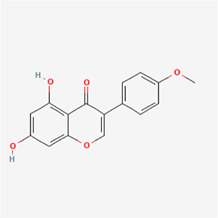	*Sojae Semen Praeparatum, Spatholobus Suberectus Dunn*
	formononetin	485-72-3	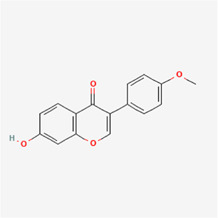	*Hedysarum Multijugum Maxim., licorice, Radix Puerariae*
	daidzein	486-66-8	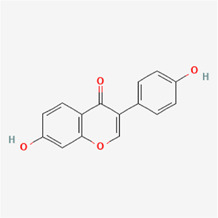	*Hedysarum Multijugum Maxim., Radix Puerariae, Sojae Semen Praeparatum*
	kaempferol	520-18-3	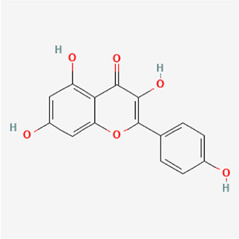	*Carthami Flos, Caryophylliflos, Astragalus membranaceus*
	icariin	489-32-7	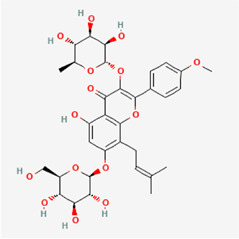	*Epimrdii Herba*
	puerarin	3681-99-0	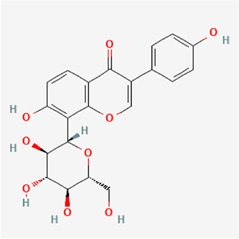	*Radix Bupleuri, Radix Puerariae*
	rutin	153-18-4	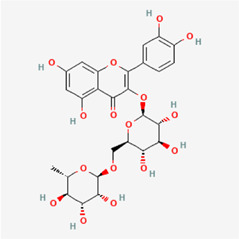	*Carthami Flos, licorice, Ephedra Herba, Hedysarum Multijugum Maxim*
Stilbenoids	resveratrol	501-36-0	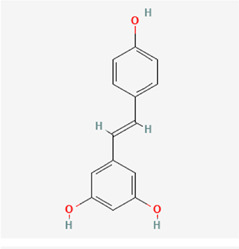	*Polygoni Cuspidati, Rhizoma Et Radix, Mori Cortex*
	polydatin	27208-80-6	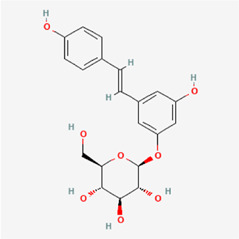	*Polygoni Cuspidati Rhizoma Et Radix*
Terpenoids	notoginsenoside R1	80418-24-2	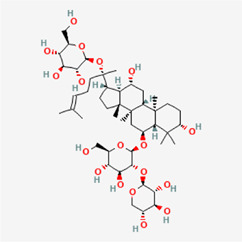	*Panacis Japonici Rhizoma*
	tanshinone IIA	568-72-9	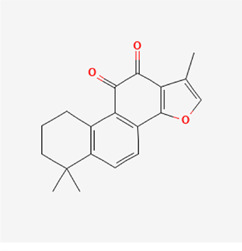	*Radix Salviae, Peucedani Radix*
	ginsenoside Rb1	41753-43-9	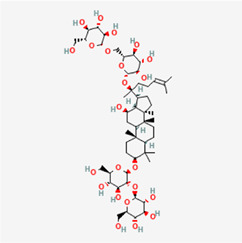	*Ginsen Radix Et Rhizoma Rubra, Panacis Japonici Rhizoma, Panax Ginseng C. A. Mey*
	ginsenoside Rb3	68406-26-8	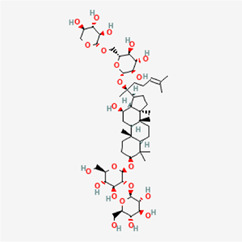	*Panax Notoginseng (Burk.) F. H. Chen Ex C. Chow*
	ginsenoside Rg1	22427-39-0	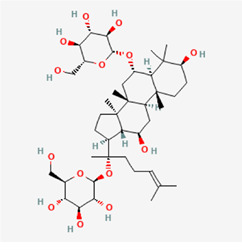	*Panacis Quinquefolii Radix*
	ginsenoside Re	52286-59-6	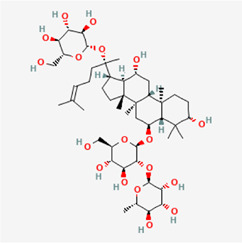	*Ginsen Radix Et Rhizoma Rubra, Panax Notoginseng (Burk.) F. H. Chen Ex C. Chow, Panax Ginseng C. A. Mey*
Miscellaneous classes	bakuchiol	10309-37-2	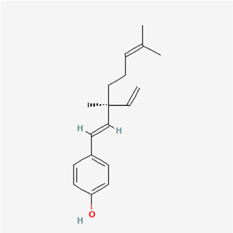	*Psoralea corylifolia Linn*

1 https://pubchem.ncbi.nlm.nih.gov/. [Accessed 10 September 2021].

2 https://old.tcmsp-e.com/tcmsp.php. [Accessed 10 September 2021].

### 3.2 Literature Retrieval Results

A total of 337 articles were obtained from various databases. After removing duplicate articles, we reviewed the abstracts and full text sequentially according to strict inclusion and exclusion criteria. Finally, a total of 20 studies from 17 articles (Li et al., 2016b; Li et al., 2016c; Gu et al., 2016; Ling et al., 2016; Liu et al., 2016; Yang et al., 2016; Cui et al., 2017; Wang et al., 2017; Li et al., 2018; Lin et al., 2018; Wu et al., 2018; Bai et al., 2019; Li et al., 2020a; Wang et al., 2020a; Wang et al., 2020b; Kazemirad and Kazerani, 2020; Jiang et al., 2021) were included in our analysis (Figure 1).

### 3.3 Study Characteristics

The characteristics of included 20 studies are provided in Table 2. The main PEs involved are genistein (n = 1), quercetin (n = 1), biochanin A (n = 1), formononetin (n = 1), kaempferol (n = 1), icariin (n = 2), puerarin (n = 1), rutin (n = 1), tanshinone IIA (n = 1), ginsenoside Rb1 (n = 6), ginsenoside Rg1 (n = 1), resveratrol (n = 2), and polydatin (n = 1). A total of 17 studies were conducted *in vivo* using ligation followed by the release of LAD to simulate MIRI, and the remaining three studies were conducted *ex vivo* using Langendroff perfusion. The author Wu B conducted both *in vivo* and *ex vivo* studies, which we labeled and distinguished with “Wu B 2018 (1)” and “Wu B 2018 (2).” All studies used infarct size as the primary efficacy index, with 12 studies using TTC single staining and 8 using Evan’s blue/TTC double staining. A total of six kinds of anesthetics were used in the studies included in this analysis: pentobarbital sodium (n = 12), thiopental sodium (n = 1), urethane (n = 1), chloral hydrate (n = 2), isoflurane (n = 3), and a mixture of xylazine and ketamine (n = 1). A total of 355 animals were enrolled for our study, including SD rats, Wistar rats, and C57BL/6J mice. Several studies have included markers of myocardial injury such as levels of cardiac troponin I (cTnI) (n = 5) and creatine kinase (CK) (n = 2) as outcome indicators in their analyses. Seven studies performed echocardiography on animals and reported LVEF and LVFS. All articles were written in English. Only one study is from Iran, the rest were conducted in China.

**TABLE 2:** Characteristics of the included studies.

Study (years)	State	Species (Sex, Wight, n = Treatment/Control Group)	Methods of i/R	I/R Duration	Anesthetics	Treatment Group			Control group	Outcome Index	Staining Method
						PE Type	Dosage	Approach (Time)			
Bai YJ 2019	China	Sprague-Dawley rats (male, 220–250 g, n = 18/6)	*in vivo*	30 min/2 h	pentobarbital sodium	biochanin A	12.5, 25, 50 mg/kg/d	intragastric administration (before I/R)	I/R	IFS	TTC staining
Cui YC 2017	China	Sprague-Dawley rats (male, 230–270 g, n = 18/6)	*in vivo*	30 min/1.5 h	urethane	kaempferol	2.5, 5 or 7.5 mg/kg/h	intravenous infusion (start from 30 min before ischemia until the end of reperfusion)	I/R + NS	IFS, cTnI, HR	Evan’s blue/TTC staining
Gu M 2016	China	Sprague-Dawley rats (male, 250–300 g, n = 18/6)	*in vivo*	30 min/1 h	pentobarbital sodium	genistin	20, 40, 60 mg/kg	intragastric administration (before I/R)	I/R	IFS, CK	TTC staining
Jiang LJ 2021 (1)	China	C57BL/6J mice (male, n = 4/4)	*in vivo*	30 min/24 h	pentobarbital sodium	ginsenoside Rb1	50 mg/kg	i.p. (before I/R)	I/R	IFS	TTC staining
Jiang LJ 2021 (2)	China	C57BL/6J mice (male, n = 4/4)	*in vivo*	30 min/24 h	pentobarbital sodium	ginsenoside Rb1	50 mg/kg	i.v. (at the onset of reperfusion)	I/R	IFS	TTC staining
Jiang LJ 2021 (3)	China	C57BL/6J mice (male, n = 4/4)	*in vivo*	30 min/24 h	pentobarbital sodium	ginsenoside Rb1	50 mg/kg	i.p. (after reperfusion)	I/R	IFS	TTC staining
Li CY 2020	China	Sprague-Dawley rats (male, 230–250 g, n = 18/6)	*in vivo*	45min/2 h	chloral hydrate	ginsenoside Rb1	20、40、80 mg/kg	i.p. (before IR)	I/R	IFS	TTC staining
Li GH 2016	China	Sprague-Dawley rats (male, 220–250 g, n = 6/6)	*in vivo*	30 min/2 h	pentobarbital sodium	ginsenoside Rb1	40 mg/kg	intravenous injection (before reperfusion)	I/R + DMSO	IFS, HR	TTC staining
Li L 2018	China	Sprague-Dawley rats (male, 240–260 g, n = 6/6)	*in vivo*	30 min/1.5 h	pentobarbital sodium	ginsenoside Rg1	5 mg/kg/h	intravenous infusion (start from 30 min before ischemia until the end of reperfusion)	I/R + NS	IFS, cTnI	Evan’s blue/TTC staining
Li Q 2016	China	Sprague-Dawley rats (male, 210–250 g, n = 26/13)	*in vivo*	30 min/2 h	pentobarbital sodium	tanshinone IIA	10 mg/kg, 20 mg/kg	intravenous injection (before I/R)	I/R	IFS	Evan’s blue/TTC staining
Ling YN 2016	China	C57BL/6J mice (male, 20–25 g, n = 4/4)	*in vivo*	30 min/2 h	xylazine and ketamine	polydatin	7.5 mg/kg	i.p. (after reperfusion)	I/R + NS	IFS, LVEF, LVFS	Evan’s blue/TTC staining
Wang D 2017	China	Sprague-Dawley rats (male, 250–300g, n = 18/6)	*in vivo*	30 min/2 h	pentobarbital sodium	kaempferide	0.1 mg/kg, 0.3 mg/kg, 1 mg/kg	unclear (before I/R)	I/R	IFS, CK, LVEF, LVFS	TTC staining
Wang DS 2020	China	Sprague-Dawley rats (male, 250–280 g, n = 20/10)	*in vivo*	60 min/24 h	isoflurane	formononetin	10 mg/kg, 30 mg/kg	intraperitoneal injection (when reperfusion started)	I/R + vehicle	IFS, cTnI, LVEF, LVFS	Evan’s blue/TTC staining
Wang ZK 2020	China	C57BL/6J mice (male, 20–25 g, n = 6/6)	*in vivo*	30 min/24 h	pentobarbital sodium	puerarin	100 mg/kg	intraperitoneal injection (before reperfusion)	I/R	IFS, LVEF, LVFS	Evan’s blue/TTC staining
Wu B 2018 (1)	China	Sprague-Dawley rats (male, 220–250 g, n = 8/8)	*in vivo*	30 min/24 h	isoflurane	Icariin	60 mg/kg	intragastric administration (after I/R)	I/R + DMSO/PBS	IFS, LVEF, LVFS	Evan’s blue/TTC staining
Wu B 2018 (2)	China	C57BL/6J mice (male, 20–25 g, n = 8/8)	*ex vivo*	40 min/1 h	pentobarbital sodium	Icariin	10 μmol/L	Langendorff perfusion (during reperfusion)	I/R	IFS, HR	TTC staining
Lin Q 2018	China	Sprague-Dawley rats (male, 200–250 g, n = 9/3)	*in vivo*	30 min/24 h	pentobarbital sodium	rutin	80 mg/kg, 40 mg/kg, 20 mg/kg	i.p. (before I/R)	I/R + NS containing 0.5% CMC-Na	IFS, cTnI, LVEF, LVFS	TTC staining
Liu XY 2016	China	C57/BL6 mice (male, 20–22 g, n = 6/6)	*in vivo*	30 min/24 h	isoflurane	quercetin	250 mg/kg	intragastric administration (before I/R)	I/R + DMSO	IFS, LVEF, LVFS	Evan’s blue/TTC staining
Yang L 2016	China	Sprague-Dawley rats (male, n = 10/10)	*ex vivo*	30 min/1 h	chloral hydrate	resveratrol	10 μmol/L	Langendorff perfusion (during reperfusion)	I/R	IFS	TTC staining
Kazemirad H 2020	Iran	Wistar rats (male, 250–300 g, n = 10/10)	*ex vivo*	30 min/2 h	thiopental sodium	resveratrol	10 μmol/L	Langendorff perfusion (before I/R and during reperfusion)	I/R	IFS, cTnI, HR	TTC staining

PE: phytoestrogen; LAD: left anterior descending; IFS: infarct size; TTC: tetrazolium chloride; HR: heart rate; CK: creatine kinase; cTnI: cardiac troponin I; NS: normal saline; i. p.: intraperitoneal injection; i. v, intravenous injection; DMSO: dimethyl sulfoxide; LVEF: left ventricular ejection fraction; LV, left ventricular ejection fraction; LVFS: left ventricular fractional shortening.: left ventricular fractional shortening.

### 3.4 Quality Appraisal

We used the SYRCLE’s RoB tool to score the quality of each study. Table 3 shows the information on methodological quality. As shown in Table 3, six studies scored five points, and four studies scored only two points, which indicate reliable data but lower quality of studies. Ten studies mentioned randomized groupings, but all were silent on the specific randomization method. All studies did not describe how allocation concealment is performed.

**TABLE 3:** Quality assessment of included studies.

Study (years)	A	B	C	D	E	F	G	H	I	J	Total
Bai YJ 2019	?	Y	?	Y	N	Y	N	Y	?	Y	5
Cui YC 2017	?	Y	?	?	N	Y	N	Y	?	N	3
Gu M 2016	?	Y	?	Y	N	Y	N	N	?	Y	4
Jiang LJ 2021 (1)	?	?	?	?	N	Y	N	?	?	Y	2
Jiang LJ 2021 (2)	?	?	?	?	N	Y	N	?	?	Y	2
Jiang LJ 2021 (3)	?	?	?	?	N	Y	N	?	?	Y	2
Li CY 2020	?	Y	?	?	N	Y	N	?	?	Y	3
Li GH 2016	?	Y	?	Y	N	Y	N	N	?	Y	4
Li L 2018	?	Y	?	Y	N	Y	N	Y	?	Y	5
Li Q 2016	?	Y	?	Y	N	Y	N	Y	?	Y	5
Ling YN 2016	?	Y	?	?	N	Y	N	?	?	Y	3
Wang D 2017	?	Y	?	Y	N	Y	N	Y	?	Y	5
Wang DS 2020	?	Y	?	?	N	Y	N	?	?	Y	3
Wang ZK 2020	?	Y	?	Y	N	Y	N	N	?	Y	4
Wu B 2018 (1)	?	Y	?	?	N	Y	N	?	?	Y	3
Wu B 2018 (2)	?	Y	?	Y	N	Y	N	?	?	Y	4
Lin Q 2018	?	Y	?	Y	N	Y	N	Y	?	Y	5
Liu XY 2016	?	Y	?	Y	N	Y	N	Y	?	Y	5
Yang L 2016	?	?	?	?	N	Y	N	?	?	Y	2
Kazemirad H 2020	?	Y	?	?	N	Y	N	Y	?	Y	4

Y: yes (low risk of bias); N: No (high risk of bias); ? unclear bias.(A) sequence generation; (B) baseline characteristics; (C) allocation concealment; (D) random housing; (E) blinding investigators; (F) random outcome assessment; (G) blinding outcome assessor; (H) incomplete outcome data; (I) selective outcome reporting; (J) other sources of bias.

### 3.5 Outcome Measures

#### 3.5.1 Infarct Size

**FIGURE 2:**
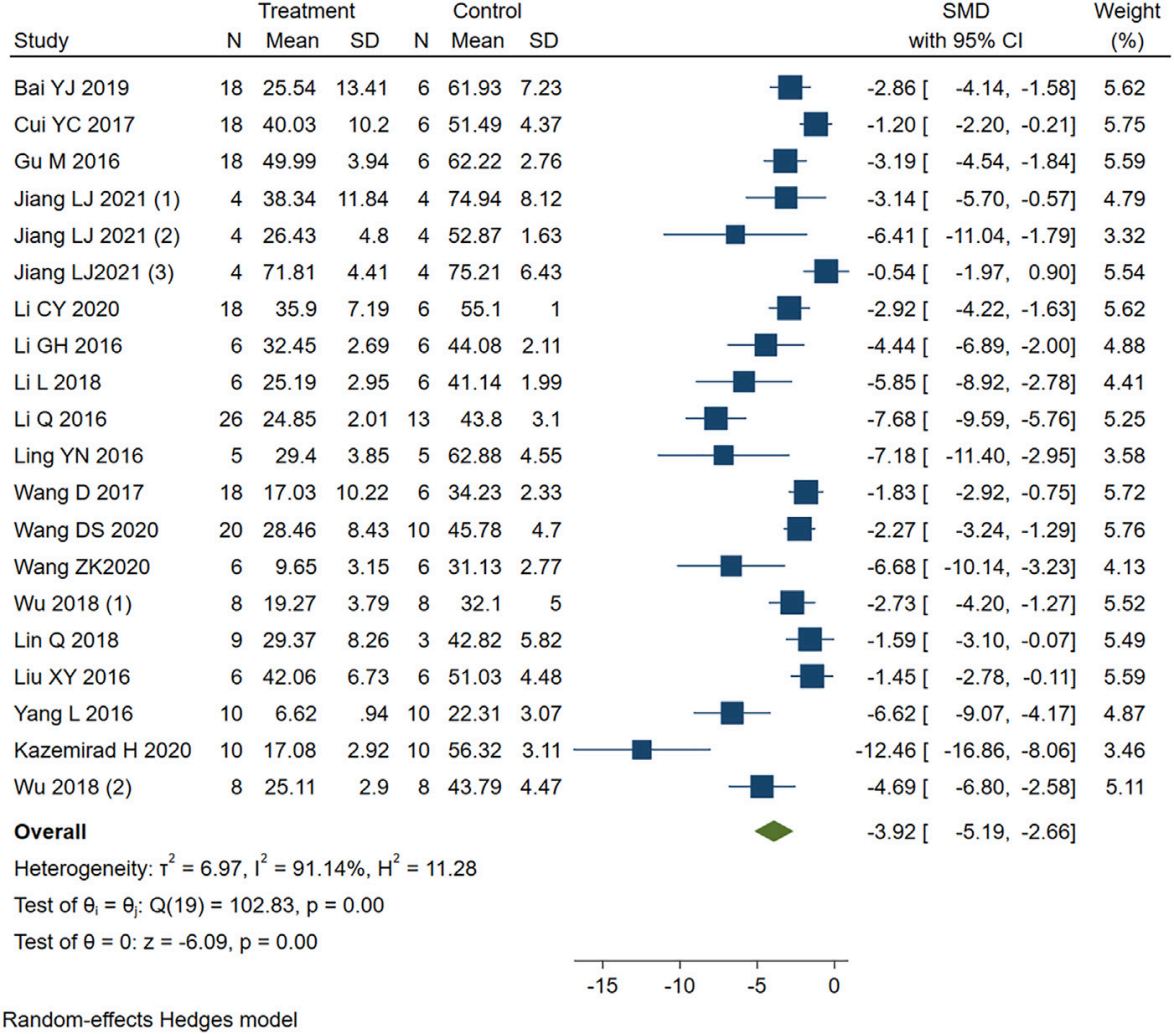
Forest plot to study the effect of PE on infarct size. PE reduced the myocardial infarct size in MIRI animals compared with the control group (SMD = −3.92, 95%CI: −5.19 to −2.66, *p* < 0.001). The dark squares represent the standardized mean difference (SMD) for each study. The diamonds represent the pooled SMD. 95% of the CIs are indicated by lines. The analysis was conducted using a fixed-effects model.

Studies that reported infarct size were analyzed using a random-effects model. Figure 2 showed that PE significantly reduced the myocardial infarct size in MIRI animals compared with the control group (SMD = −3.92, 95%CI: −5.19 to −2.66, *p* < 0.001). I^2^>50%, which suggests high heterogeneity. We first performed sensitivity analysis by removing each study in turn to assess the impact of this study. Then we performed the stratified analysis (Table 4) and meta-regression (Table 5) with study type, reperfusion time, route of administration, animal species, staining method, or PE type as covariates. Sensitivity analysis and stratified analysis failed to find significant sources of heterogeneity, while meta-regression confirms study type as a heterogeneity source (*p* = 0.046 < 0.05). Egger’s (*p* = 0.00) (Supplementary Figure S1) and Begg’s (*p* = 1.999) (Supplementary Figure S2) tests confirmed the possible existence of publication bias. After additional adjustment for potential missing studies by nonparametric trim-and-fill analysis (Supplementary Figures S3, S4), the statistical results support the robust effect of PE treatment (SMD = -2.634, 95%CI: -4.204 to -1.065, *p* < 0.001). This result implies that PE can effectively reduce the size of myocardial infarct area in MIRI animals, which may be related to the type of animal experiment involved.

**TABLE 4:** Stratified analysis of pooled estimates of infarct size.

Pooled Estimates	No. of Studies	SMD	95% CI	*p* value	Heterogeneity
Study type					
*In vivo*	17	−3.26	−4.31, −2.21	*p* = 0.00	I^2^ = 86.12%
*Ex vivo*	3	−7.6	−12.15, −3.04	*p* = 0.01	I^2^ = 87.32%
Reperfusion duration					
1	3	−4.62	−6.54, −2.69	*p* = 0.05	I^2^ = 66.07%
1.5	2	−3.29	−7.82, −1.24	*p* = 0.00	I^2^ = 87.44%
2	7	−5.27	−8.02, −2.52	*p* = 0.00	I^2^ = 93.95%
24 h	8	−2.63	−4.14, −1.11	*p* = 0.01	I^2^ = 83.94%
Route of administration					
intragastric administration	4	−2.55	−3.30, −1.81	*p* = 0.29	I^2^ = 17.59%
intraperitoneal injection	7	−3.04	−4.86, −1.22	*p* = 0.00	I^2^ = 86.9%
intravenous injection	3	−6.44	−7.88, −5.01	*p* = 0.12	I^2^ = 0%
intravenous infusion	2	−3.29	−7.82, −1.24	*p* = 0.00	I^2^ = 87.44%
unclear	1	−1.83	−2.92, −0.75	—	—
Langendorff perfusion	3	−7.6	−12.15, −3.04	*p* = 0.01	I^2^ = 87.32%
Staining method					
Single staining	12	−4.02	−5.87, −2.16	*p* = 0.00	I^2^ = 90.11%
Double staining	8	−4	−5.93, −2.08	*p* = 0.00	I^2^ = 91.84%
PE type					
isoflavone	9	−2.76	−3.83, −1.68	*p* = 0.04	I^2^ = 80.24%
triterpene	8	−3.73	−5.44, −2.02	*p* = 0.00	I^2^ = 85.14%
Stilbenes	3	−8.47	−12.08, −4.87	*p* = 0.07	I^2^ = 65.96%
Animal species					
Rats	13	−3.94	−5.08, −2.80	*p* = 0.00	I^2^ = 81.82%
Mice	7	−3.444	−5.384, −1.504	*p* = 0.00	I^2^ = 93.72%
Anesthetics type					
pentobarbital sodium	12	−3.75	−4.98, −2.51	*p* = 0.00	I^2^ = 80.95
Urethane	1	−1.2	−2.20, −0.21	—	—
chloral hydrate	2	−4.62	−8.23, −1.01	*p* = 0.01	I^2^ = 85.35
xylazine and ketamine	1	−7.18	−11.40, −2.95	—	—
isoflurane	3	−2.15	−2.84, −1.46	*p* = 0.42	I^2^ = 0
thiopental sodium	1	−12.46	−16.86, −8.06	—	—

**TABLE 5:** Meta-regression analysis.

Heterogeneity Factor	Coefficient	Std. Err	Z Value	*p* Value	95% CI
study type	−6.28513	2.811002	−2.24	0.025	−11.79459, −0.7756678
reperfusion duration	0.1080382	0.0830519	1.3	0.193	−0.0547405, 0.270817
route of administration	−0.8261537	0.5889316	−1.4	0.161	−1.980438, 0.328131
animal species	−1.428073	1.7573	−0.81	0.416	−4.872319, 2.016172
staining method	−0.3233558	1.15108	−0.28	0.779	−2.579432, 1.93272
PE type	9.462195	5.151416	1.84	0.066	−0.6343956, 19.55879

#### 3.5.2 Markers of Myocardial Injury

**FIGURE 3:**
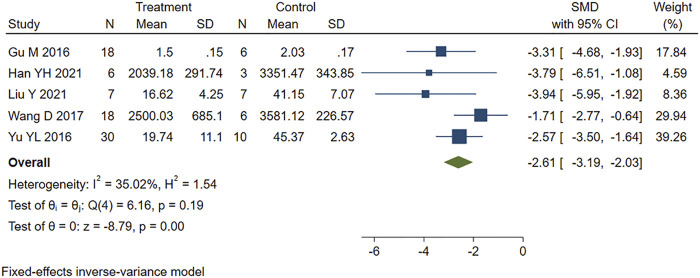
Forest plot to study the effect of PE on creatine kinase (CK). PE reduced serum CK levels in MIRI animals compared to control group (SMD = −2.61, 95% CI: −3.19 to −2.03, *p* < 0.001). The dark squares represent the standardized mean difference (SMD) for each study. The diamonds represent the pooled SMD. 95% of the CIs are indicated by lines. The analysis was conducted using a fixed-effects model.

Five studies that reported serum CK levels were analyzed using a fixed-effects model. Figure 3 shows that PE treatment was associated with significantly lower CK levels in MIRI animals (SMD = −2.61, 95% CI: −3.19 to −2.03, *p* < 0.001).

**FIGURE 4:**
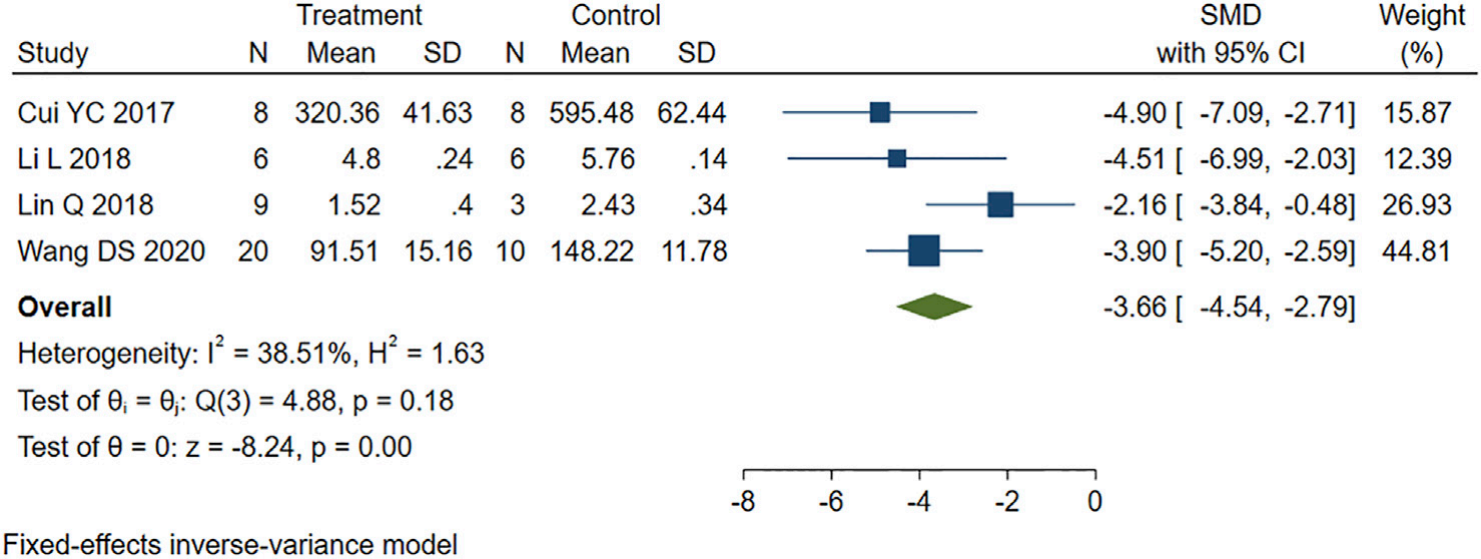
Forest plot to study the effect of PE on cardiac troponin I (cTnI). PE reduced serum cTnI levels in MIRI animals compared to control group (SMD = −3.66, 95% CI: −4.54 to −2.79, *p* < 0.001). The dark squares represent the standardized mean difference (SMD) for each study. The diamonds represent the pooled SMD. 95% of the CIs are indicated by lines. The analysis was conducted using a fixed-effects model.

The five studies that reported cTnI levels showed high heterogeneity (I2 = 82.24%, *p* = 0.00). We performed a sensitivity analysis by systematically excluding each study. One study (Kazemirad and Kazerani, 2020) was identified as a source of heterogeneity and was therefore removed. The other four studies were analyzed using fixed-effects models. The results showed that PE treatment significantly reduced the cTnI level in MIRI animals compared to the control group (SMD = −3.66, 95% CI: −4.54 to −2.79, *p* < 0.001) (Figure 4).

The statistical results suggest that the serum CK and cTnI levels in the PE group were significantly lower than those in the control group, indicating that PE treatment can effectively alleviate the myocardial damage caused by MIRI.

#### 3.5.3 Indicators of Cardiac Function

**FIGURE 5:**
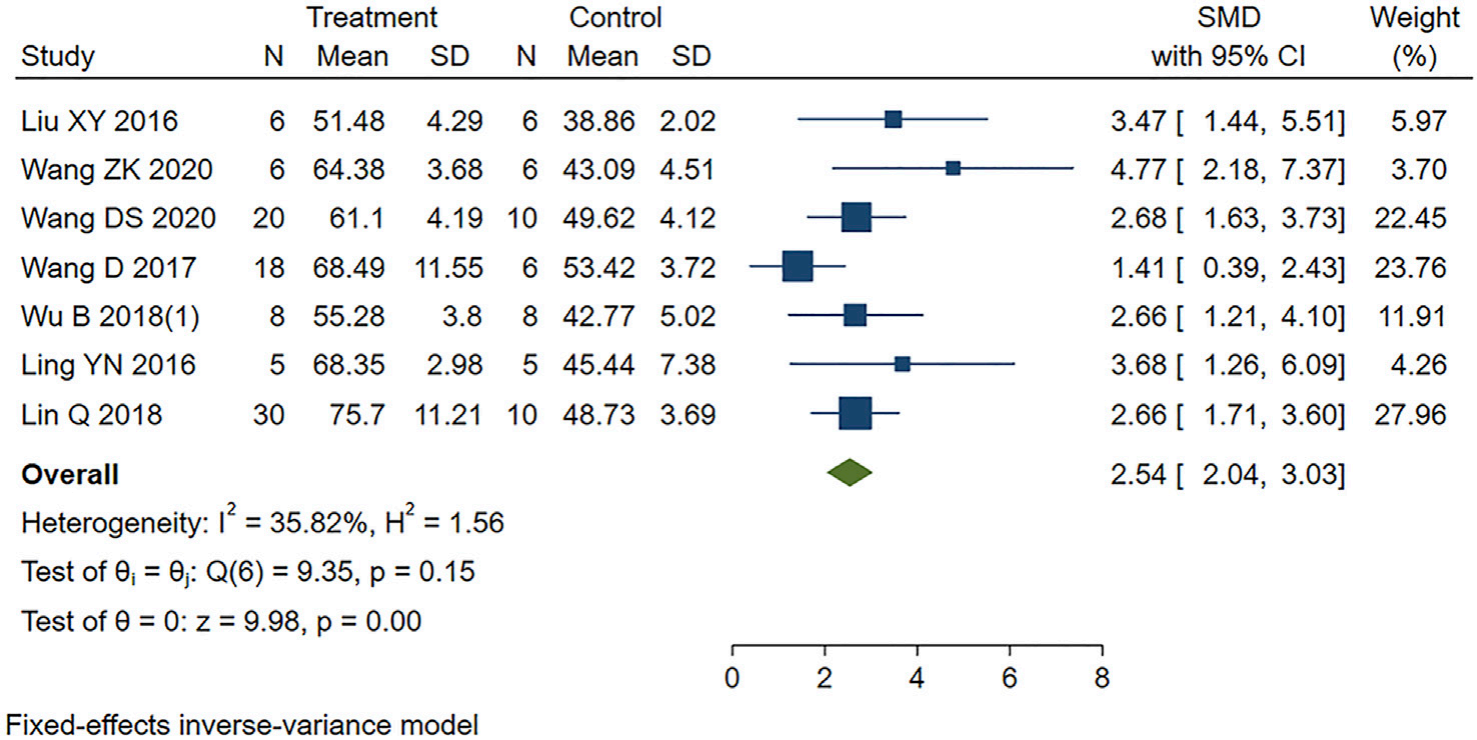
Forest plot to study the effect of PE on left ventricular ejection fraction (LVEF). PE improved LVEF in MIRI animals compared to control group (SMD = 2.54, 95% CI: 2.04 to 3.03, *p* < 0.001). The dark squares represent the standardized mean difference (SMD) for each study. The diamonds represent the pooled SMD. 95% of the CIs are indicated by lines. The analysis was conducted using a fixed-effects model.

Seven studies appropriately reported the effect of PE on LVEF in MIRI animals. Analysis using a fixed-effects model showed that PE significantly increased LVEF (SMD = 2.54, 95% CI: 2.04 to 3.03, *p* < 0.001) (Figure 5).

**FIGURE 6:**
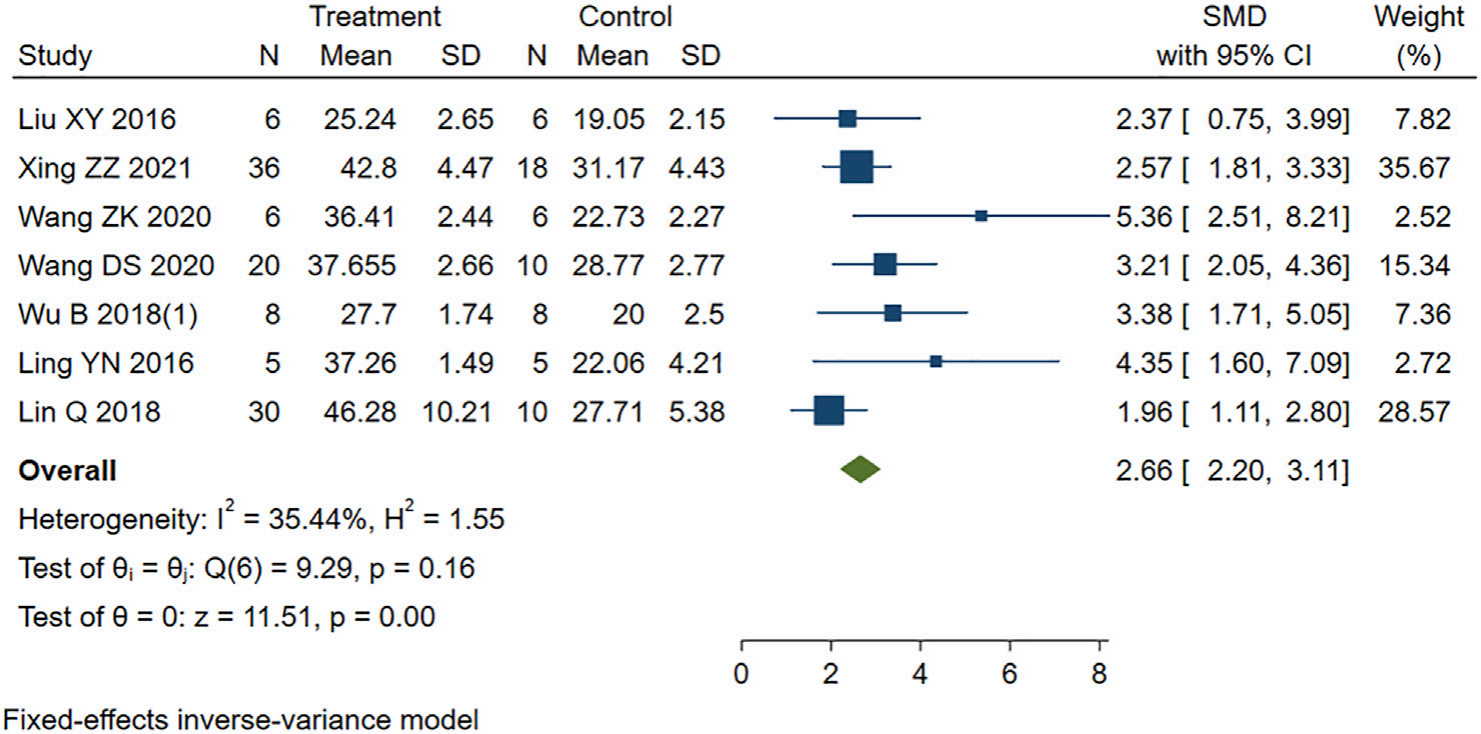
Forest plot to study the effect of PE on left ventricular fractional shortening (LVFS). PE improved LVFS in MIRI animals compared to control group (SMD = 2.66, 95% CI: 2.20 to 3.11, *p* < 0.001). The dark squares represent the standardized mean difference (SMD) for each study. The diamonds represent the pooled SMD. 95% of the CIs are indicated by lines. The analysis was conducted using a fixed-effects model.

Similarly, moderate heterogeneity (I2 = 48.38%, *p* < 0.001) was found in the eight studies reporting LVFS in our study. We removed one study identified (Wang et al., 2017) by sensitivity analysis as the heterogeneous source. The remaining seven studies were included in the analysis using a fixed-effects model. Figure 6 showed that PE treatment led to higher LVFS compared to the control group (SMD = 2.66, 95% CI: 2.20 to 3.11, *p* < 0.001).

The statistical results showed that the difference between PE group and control group was statistically significant. PE treatment can improve cardiac function in MIRI animals.

#### 3.5.4 Heart Rate

**FIGURE 7:**
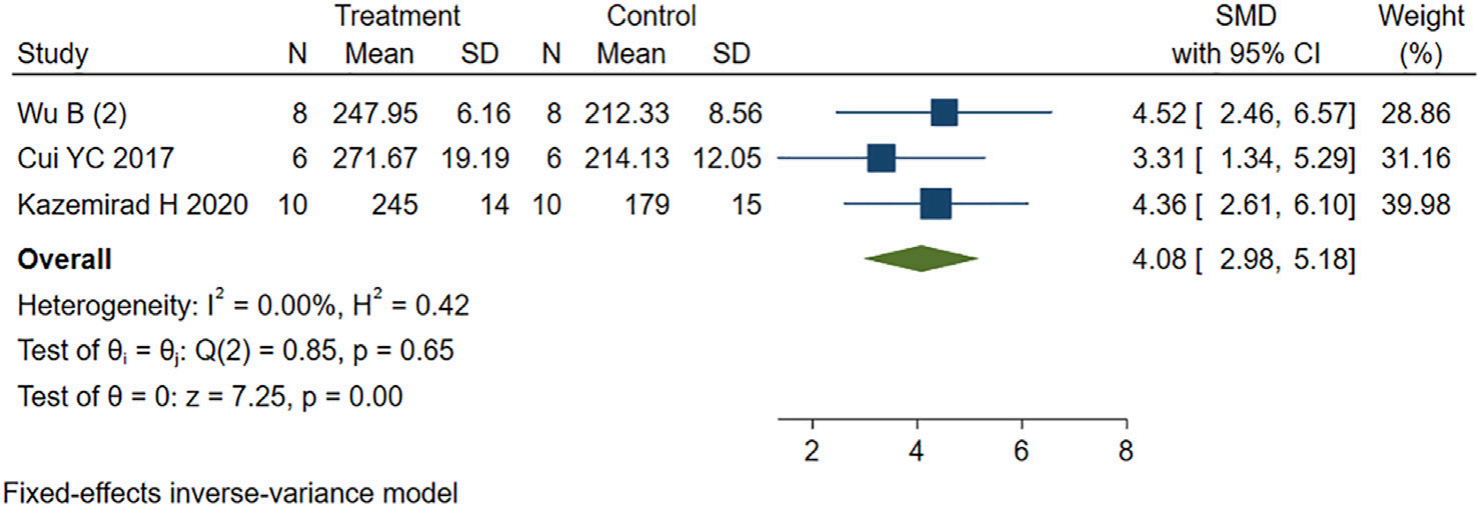
Forest plot to study the effect of PE on heart rate. PE reduced heart rate in MIRI animals compared to control group (SMD = 4.08 95% CI: 2.98 to 5.18, *p* < 0.001). The dark squares represent the standardized mean difference (SMD) for each study. The diamonds represent the pooled SMD. 95% of the CIs are indicated by lines. The analysis was conducted using a fixed-effects model.

Four other studies reported the effect of PE on heart rate variability in MIRI animals. Sensitivity analysis identified and removed one study (Kazemirad and Kazerani, 2020) that was considered a source of heterogeneity. The remaining studies were included in the analysis using a fixed-effects model. The new combined effect size determined the HR lowering effect of PE treatment on MIRI animals (SMD = 4.08 95% CI: 2.98 to 5.18, *p* < 0.001) (Figure 7), as did the total combined effect size. This indicates that the PE treatment group can reduce the heart rate of MIRI animals compared to the control group, and the difference is statistically significant.

## 4 Discussion

The incidence of CHD increases rapidly in postmenopausal women, with declining estrogen levels being the main cause (Barrett-Connor, 2013). Myocardial infarction (MI) is milder and occurs later in women than in men of the same age (Regitz-Zagrosek and Kararigas, 2017). These sex differences disappear after natural or surgical menopause (ovariectomy) (McSweeney et al., 2016), or under conditions impairing ovarian function and thus estrogen production (Barton, 2013). The effects of estrogen on MIRI are partly attributed to the potent anti-inflammatory (Wang et al., 2006), antioxidant (Kim et al., 1996), and mitochondrial protective properties (Zhai et al., 2000; Wang et al., 2019) of estrogen. There have been some studies demonstrating that elevated estrogen levels are likely to be the cause of PE’s efficacy for MIRI in ovariectomized rats (Zhai et al., 2001; Hao et al., 2011; Tang et al., 2016). PEs are found in many Chinese herbs such as ginseng (Renshen in Chinese), salvia (Danshen in Chinese), geranium (Gegen in Chinese), and safflower (Honghua in Chinese), which are often included in prescriptions for the Chinese medicine treatment of CHD (Li and Zhao, 2009; Chiu et al., 2011; Huang et al., 2016; Li et al., 2020b; Zhang et al., 2021a; Zhang et al., 2021b). In recent years, researchers have made significant progress in the treatment of MIRI with PEs and have published a large number of research results. However, these results have not been systematically analyzed.

### 4.1 Summary of Evidence

A total of 355 animals were included in our study, 222 of which were treated with PE. As a class of cardioprotective agent, PEs exerted significant anti-MIRI effects in animal models, mainly in reducing infarct size (*p* < 0.001), mitigating myocardial injury (*p* < 0.001), improving cardiac function (*p* < 0.001) and lowering heart rate (*p* < 0.001). Moreover, this cardioprotective effect was not limited by factors such as phytoestrogen species, rodent species, methods of I/R, I/R duration, or anesthetics, which were found by stratified analysis, sensitivity analysis, and regression analysis of different outcome indicators.

### 4.2 Molecular Mechanisms

Prior to conducting a clinical trial, animal models can be used to determine the effectiveness of a drug or procedure and to explore its mechanisms. Through a comprehensive search of various databases, the specific mechanisms of PE against MIRI can be summarized as follows.

1. Mitochondrial pathway. The primary mechanism we found is a disturbance in mitochondrial structure, function, and quantity. Mitochondrial structure and function are impaired by ischemia and aggravated by reperfusion (Giedt et al., 2012; Consolini et al., 2017; Ham and Raju, 2017; Anzell et al., 2018). MIRI causes impairment of the mitochondrial respiratory chain leading to abnormal energy metabolism (Leist et al., 1997; Tahrir et al., 2019), and also causes excessive accumulation of reactive oxygen species (ROS) producing oxidative stress (Li and Jackson, 2002; Lesnefsky et al., 2017). PE can suppress mitochondrial swelling and reduce the number of fragmented mitochondria in terms of retaining mitochondrial structure (Zhai et al., 2001). As for the involvement in energy metabolism, PEs has been shown to inhibit the activation RhoA/ROCK signaling (He et al., 2014; Cui et al., 2017; Yan et al., 2021) as well as activate the mK_ATP_ channels ((Colareda et al., 2020), (Colareda et al., 2021)), thus promote ATP production. PE also directly or indirectly regulates the activity of mitochondrial complex. For example, PE has been found not only to reduce NADH dehydrogenase activity and inhibit mitochondrial complex I activity, but also to maintain mitochondrial complex V activity. (Jiang et al., 2021; Yan et al., 2021). Meanwhile, PE can further ameliorate oxidative stress injury in cardiomyocytes by regulating silent information regulator 1 (SIRT1) and thus downstream targets such as peroxlsome proliferator-activated receptor-γ coactlvator-1α (PGC-1α) (Feng et al., 2016; Zhong et al., 2019) and forkhead box O 1 (FOXO1) (Wu et al., 2018). Furthermore, it has been suggested that PE’s effect on mitochondrial function is dosage-dependent (Barton, 2013). It works as an antioxidant and enhances mitochondrial biogenesis at low dosages, but it also functions as a pro-oxidant and impairs mitochondrial function at high dosages (Roca et al., 2014). For example, Genistein has been reported to promote cell proliferation at low concentrations (0.1–10M) and inhibit cell proliferation at high concentrations (above 10M) (Matsumura et al., 2005; Seo et al., 2006).
2. Inflammation and immunity. According to our results and analysis, inflammation and immunity have been perceived as significant markers of MIRI injury. The occurrence of I/R promotes the production of inflammatory mediators and chemokines, thus promoting the adhesion and accumulation of leukocytes in the vascular endothelium (Phillipson and Kubes, 2011); at the same time, cardiomyocytes produce large amounts of inflammatory factors (IL6, TNF-α, IL-1β) (Poynter et al., 2011), which further amplify the inflammatory response and eventually induce apoptosis in cardiomyocytes (Slegtenhorst et al., 2014). PEs can inhibit the activation of nuclear factor-κ-gene binding (NF-κB) by regulating its upstream pathway proteins toll-like receptor 4 (TLR4), JunNterminal kinase (JNK), or SIRT1((Bai et al., 2019), (Wang et al., 2020b), (Kim et al., 2009; Ma et al., 2014)). It has additionally been shown that PEs can restrain the activation of NLRP3 inflammasome through multiple pathways while inhibiting the maturation and secretion of inflammatory factors and decreasing their levels in tissues and serum (Wang et al., 2020a; Wang et al., 2020b).
3. Estrogen receptors. There are three subtypes of estrogen receptors: ERα, Erβ, and GPR30 (Deschamps and Murphy, 2009; Jia et al., 2015). Studies have shown that all ER subtypes confer cardioprotection against I/R injury both via genomic and non-genomic mechanisms ((Deschamps et al., 2010), (Deschamps and Murphy, 2009), (Zhu et al., 2020)). By interacting with ER, PEs are shown to sustain NO level by increasing the activity and expression of NO synthase (Zhai et al., 2001). Activation of ER can protect mitochondrial structural integrity and function and reduce mitochondrial autophagy ((Wang et al., 2019), (Feng et al., 2017)). Study has shown that PE significantly improved mitochondrial swelling and reduced the number of mitochondrial fragments by binding to estrogen receptors, thus maintaining the structural integrity of mitochondria. (Zhai et al., 2001).
4. Other mechanisms. In addition, our study found that the cardioprotective effects conferred by PE are associated with calcium homeostasis, ferroptosis, and endoplasmic reticulum stress. There is a concrete example that PE reduces intracellular Ca2+ level and maintains calcium homeostasis by manipulating stromal interacting molecule 1 (STIM1)-mediated store-operated calcium entry (SOCE) (Xu et al., 2019). It has been shown that PE regulates USP19/Beclin1-induced autophagy to suppress ferroptosis (Li et al., 2022). It has also been shown that multiple PEs significantly regulate the unfolded protein response (complex adaptive or pro-apoptotic signaling triggered by endoplasmic reticulum stress) associated proteins glucose-regulated protein (GRP)78, X-box binding protein (XBP)-1, cleaved activating transcription factor (ATF)-6, inositol-requiring protein-1α (IRE1α), and C/EBP-homologous protein (CHOP), in the setting of myocardial I/R injury (Kim et al., 2008; Kim et al., 2010).

**FIGURE 8:**
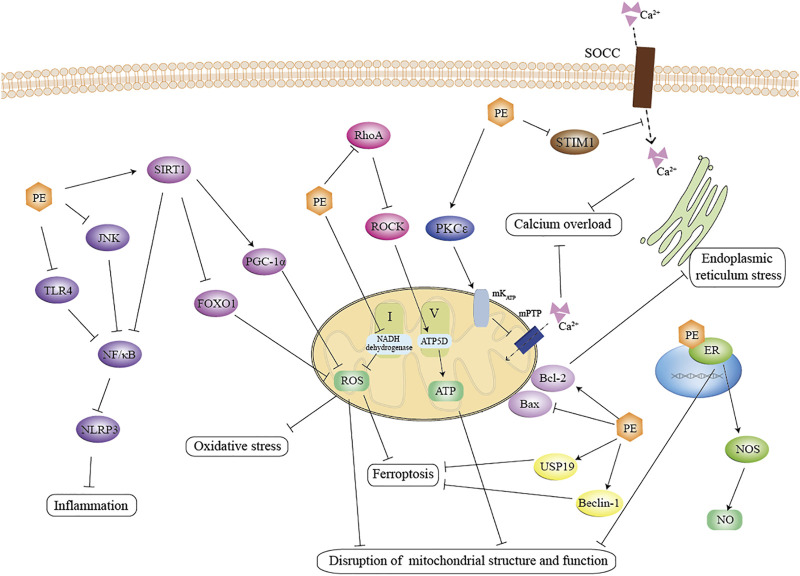
Schematic representation of the anti-MIRI effect of PE. PE suppress the inflammation by inhibiting the TLR4-NF/κB and JNK- NF/κB signaling pathways, inhibit mitochondrial oxidative stress by activating the SIRT1 pathway, and promoted the activity of the mitochondrial complex by inhibiting the RohA pathway. In addition, PE attenuated calcium overload via STIM1-mediated SOCE. Notably, PE not only maintained mitochondrial homeostasis through interaction with estrogen receptors, but also promoted NO production, which effectively exerted cardioprotective effects. TLR4: toll-like receptor four; JNK: JunNterminal kinase; NF/κB: nuclear factor-κ-gene binding; SIRT1: silent information regulator 1; FOXO1: forkhead box O; PGC-1α: proliferator-activated receptor-γ coactlvator-1α; ROS: reactive oxygen species; ROCK: RhoA/Rho-associated coiled-coil containing protein kinase; PKCε: protein kinase C; STIM1: stromal interacting molecule 1; SOCC: store-operated calcium channels; ER: estrogen receptor; NOS: nitric oxide synthase; USP19: ubiquity specific peptidase 19.

In summary, in addition to their estrogen-like cardioprotective effects as hormone replacement therapy, PEs also act through other pathways unrelated to estrogen. PE can maintain mitochondrial function and structure, alleviate oxidative stress, improve Inflammation and immune responses, and mitigate calcium overload by regulating multiple signaling pathways. The signaling pathways involved are engaged in a variety of cross-talk. The mechanisms involved are equally interactive and causal (Figure8).

## 5 Strengths and Limitations

To our knowledge, this is the first preclinical systematic review to study the cardioprotective effects of PE in MIRI animals. Not only the efficacy of PE on MIRI but also the specific mechanisms were explored in depth. There are still limitations. Systematic evaluation of animal studies is more likely to be affected by significant heterogeneity than clinical research. Phytoestrogens may lead to moderate heterogeneity depending on their form, type, dose, and route of administration, which is difficult to avoid. In addition, most of the phytoestrogens in our included studies were pre-administered and only their preventive effects and immediate efficacy were evaluated. Therefore, there is no additional evidence to further explore the long-term effects of PE and their therapeutic effects on MIRI. More large animal studies and clinical research are still needed in the future to further confirm its role in MIRI.

## 6 Conclusion

PE can play a beneficial role in MIRI by improving mitochondrial function, reducing inflammation, regulating ER, improving endoplasmic reticulum stress, and reducing ferroptosis. They are expected to be a class of cardioprotective drugs with promising development and application prospects due to their broad pharmacological effects, low toxic side effects, and high safety.

## Data Availability

The original contributions presented in the study are included in the article/Supplementary Material, further inquiries can be directed to the corresponding author.
