# Supplementary material for: A Systematic Review and Meta-Analysis of Phytoestrogen Protects Against Myocardial Ischemia/Reperfusion Injury: Pre-Clinical Evidence From Small Animal Studies
Source: Front Pharmacol. 2022 May 20;13:847748. doi: 10.3389/fphar.2022.847748 (PMC9166621; doi:10.3389/fphar.2022.847748)
Supplement: Supplementary file 1 [file DataSheet1.docx]

Supplementary Material

# Supplementary Figures

#
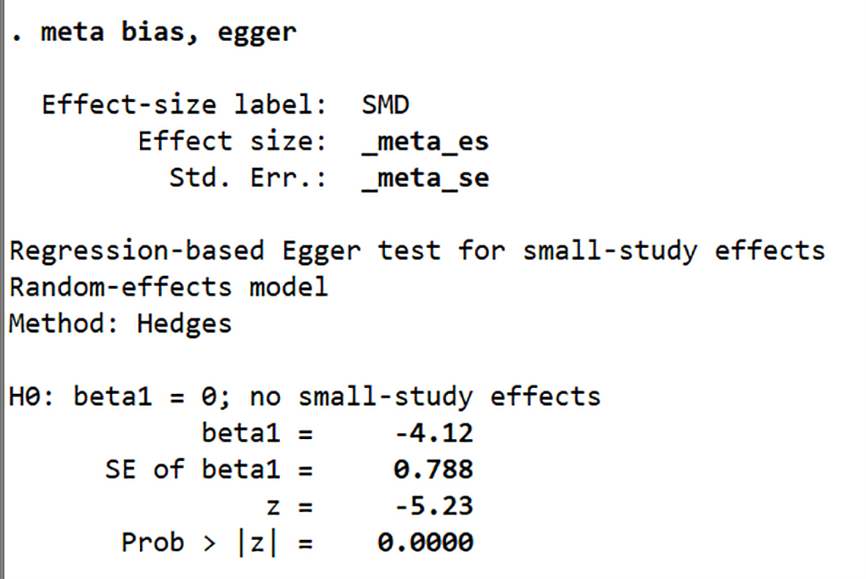


# Figure S1. The statistical result of Egger’s test.

#
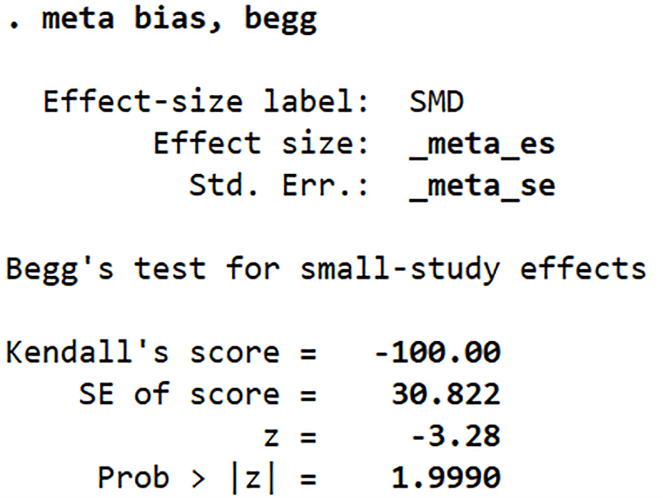


# Figure S2. The statistical result of Begg’s test.


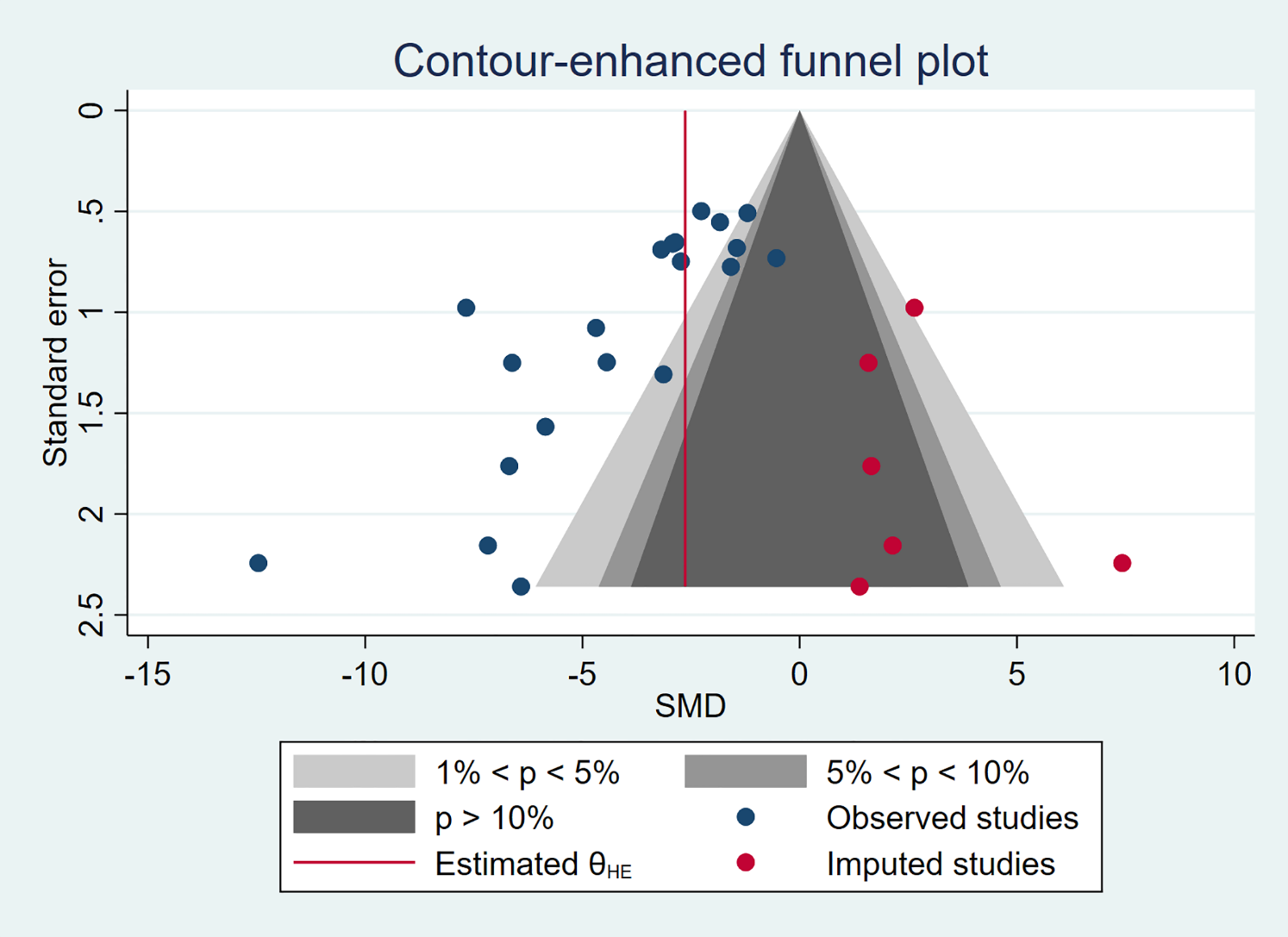


**Figure S3.** Nonparametric trim-and-fill analysis of potential missing studies.


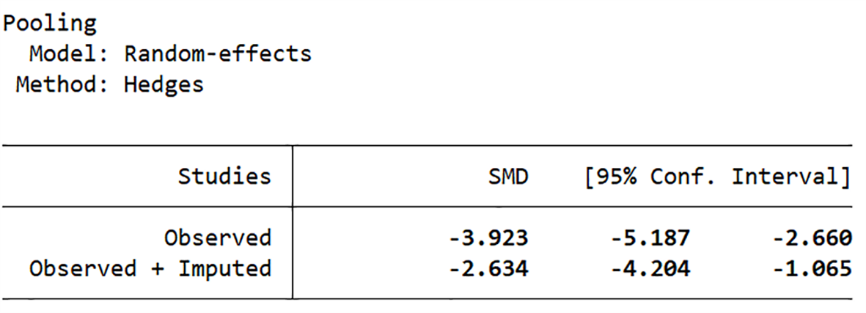


**Figure S4.** The statistical result of the nonparametric trim-and-fill analysis.
